# Supplementary material for: East Greenland ice core dust record reveals timing of Greenland ice sheet advance and retreat
Source: Nat Commun. 2019 Oct 3;10:4494. doi: 10.1038/s41467-019-12546-2 (PMC6776541; doi:10.1038/s41467-019-12546-2)
Supplement: Supplementary file 1 — Supplementary Information [file 41467_2019_12546_MOESM1_ESM.pdf]

Supplementary information

East Greenland ice core dust record reveals timing of Greenland ice sheet advance and retreat

Simonsen et al.

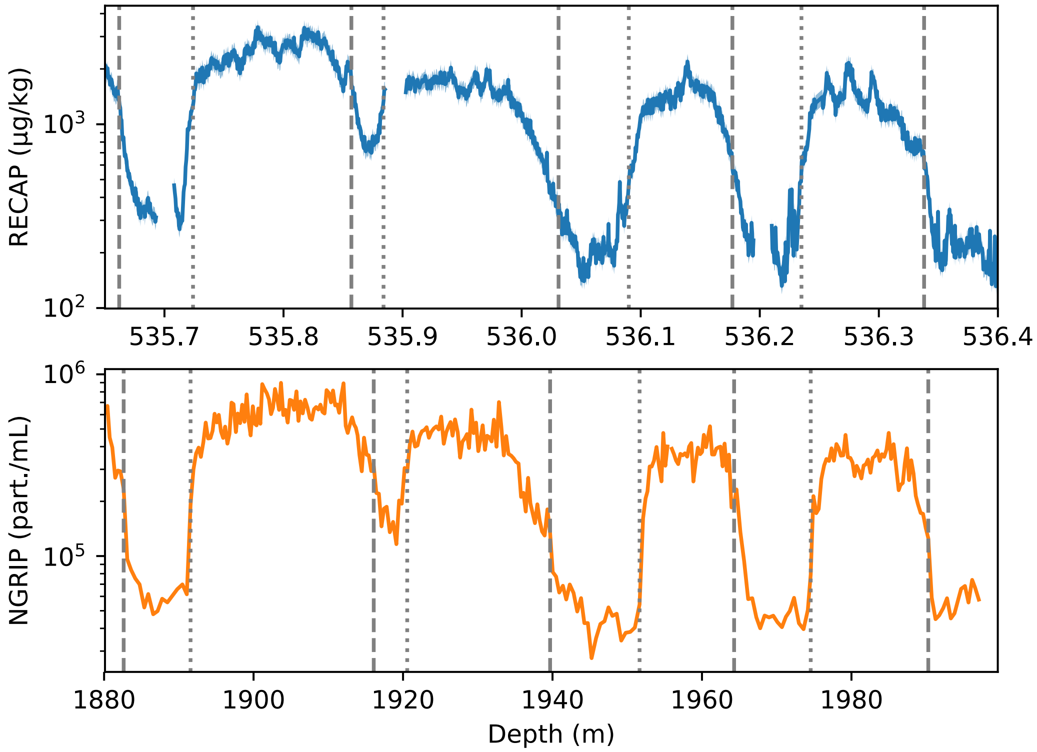

Supplementary Figure 1: **Dust record match.** Dust concentration of RECAP and NGRIP. The blue shadings around the RECAP data are 1σ errors (see Methods for details). The grey dashed and dotted lines are the tie points used for synchronising the RECAP core with the GICC05 time scale of NGRIP. The data spans from 35.1 to 28.5 ka b2k.

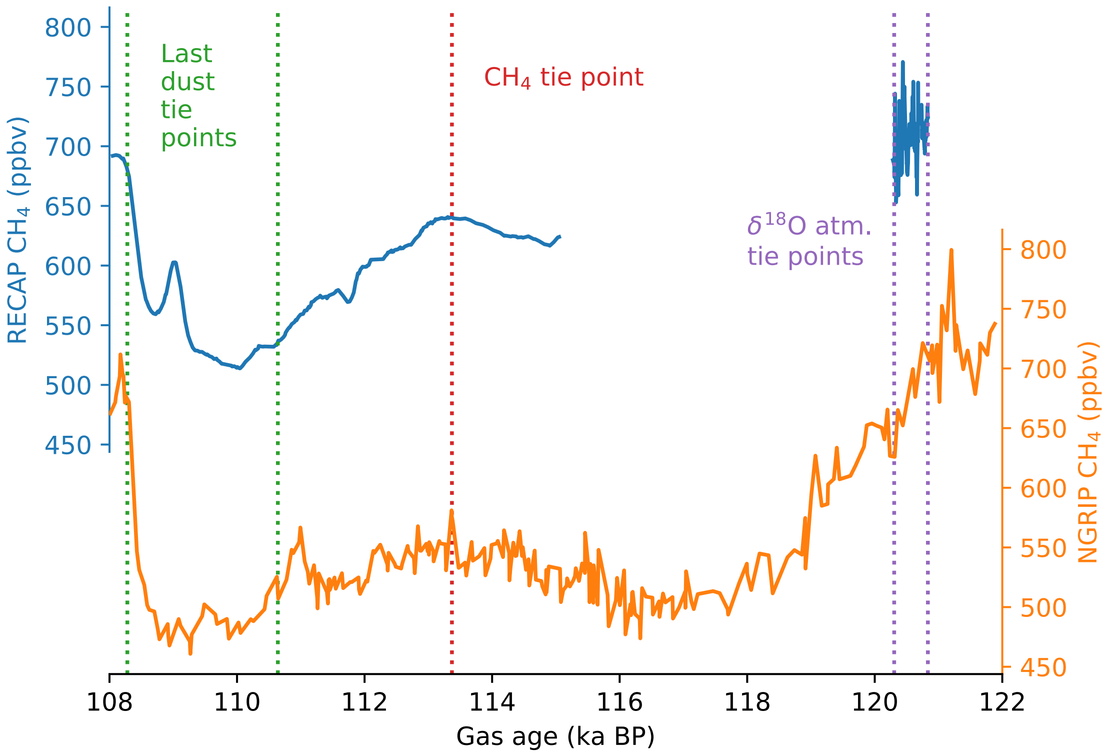

Supplementary Figure 2: **CH_4 age match.** RECAP (blue) and NGRIP (orange) CH_4 data on the GICC05modelext time scale. The 5 ka data gap in the RECAP CH_4 record corresponds to a 25 cm section of poor core quality. The two last dust transition tie points as well as the GI-25 peak CH_4 tie point and the two tie points inferred from $\delta^{18}O_{atm}$ measurements used to constrain the RECAP time scale are indicated by dashed lines.

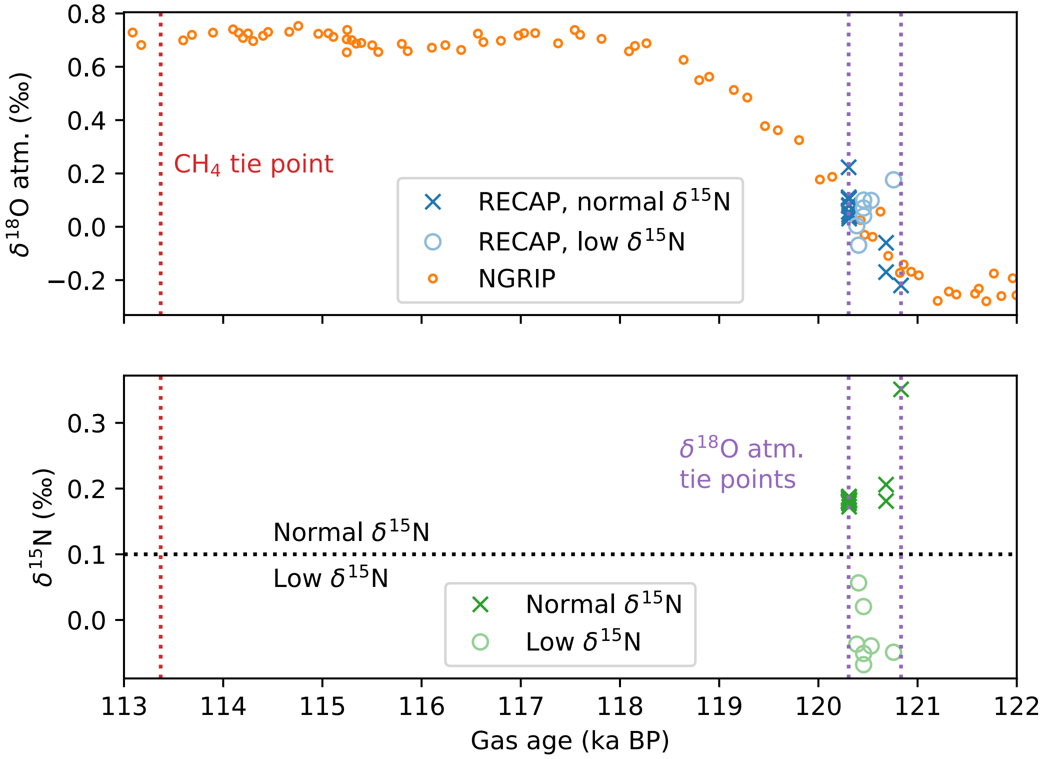

Supplementary Figure 3: **The earliest RECAP age scale tiepoints.** The top panel shows NGRIP (orange) and RECAP (blue) $\delta^{18}O_{atm}$ values on the GICC05modelext time scale. Values of RECAP $\delta^{18}O_{atm}$ that are associated with normal $\delta^{15}N$ values observed in firn columns are indicated by a cross (x), while RECAP $\delta^{18}O_{atm}$ values associated with abnormally low $\delta^{15}N$ values are indicated by a circle (o). The corresponding RECAP $\delta^{15}N$ values are shown in the lower panel. The RECAP time scale has been constructed so the trend in the RECAP $\delta^{18}O_{atm}$ values associated with normal $\delta^{15}N$ values corresponds to the trend in the NGRIP $\delta^{18}O_{atm}$ data. The three tie points derived from $\delta^{18}O_{atm}$ and CH_4 measurements are indicated by dashed lines.

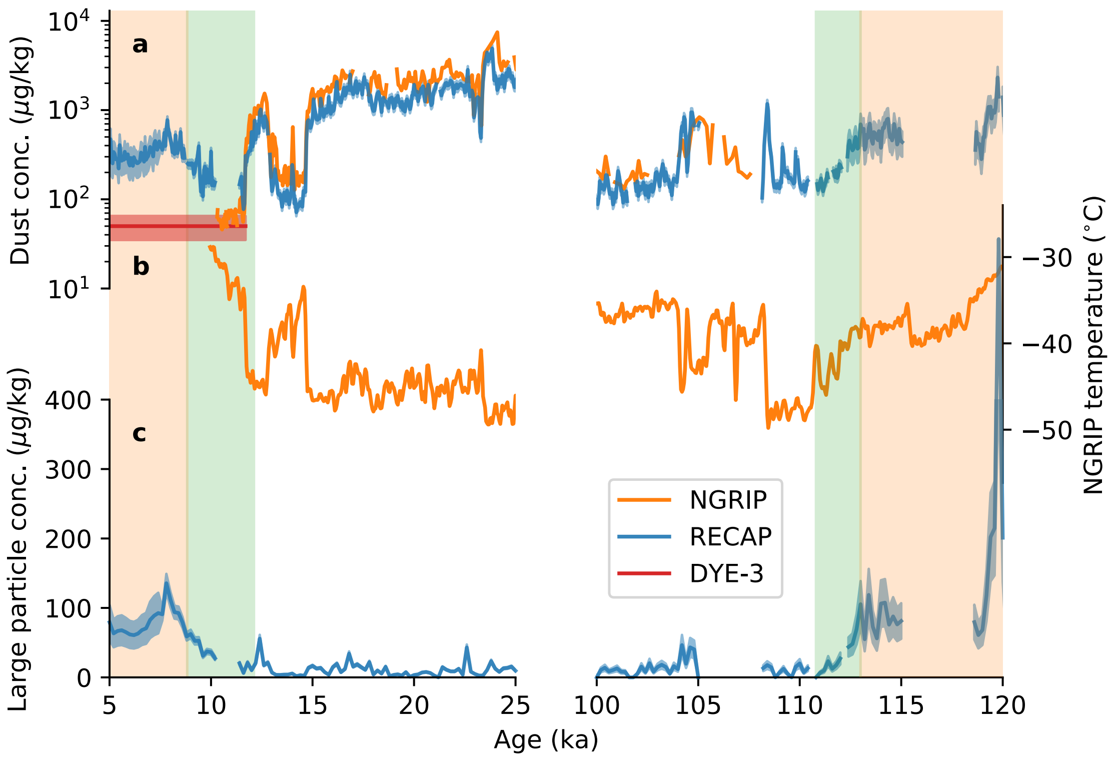

Supplementary Figure 4: **Dust and temperature records from Greenland ice cores over the glacial onset and deglaciation.** a: The total concentration of dust particles in the size range 1.25 to 10.5 µm in the RECAP and NGRIP ice cores on 50 year resolution. For DYE-3 the plot shows the average Holocene value. The coloured shadings around the curves are 1σ errors (see Methods for details). b: NGRIP temperature reconstructed from atmospheric nitrogen isotope ratios [1]. c: The large (8.13-10.5 µm) particle concentration in the RECAP ice core on 200 year resolution. The background colours indicate periods of high (orange) and low (white) large particle concentrations and the transition between the two states (green), determined by a piecewise continuous ramp fit function.

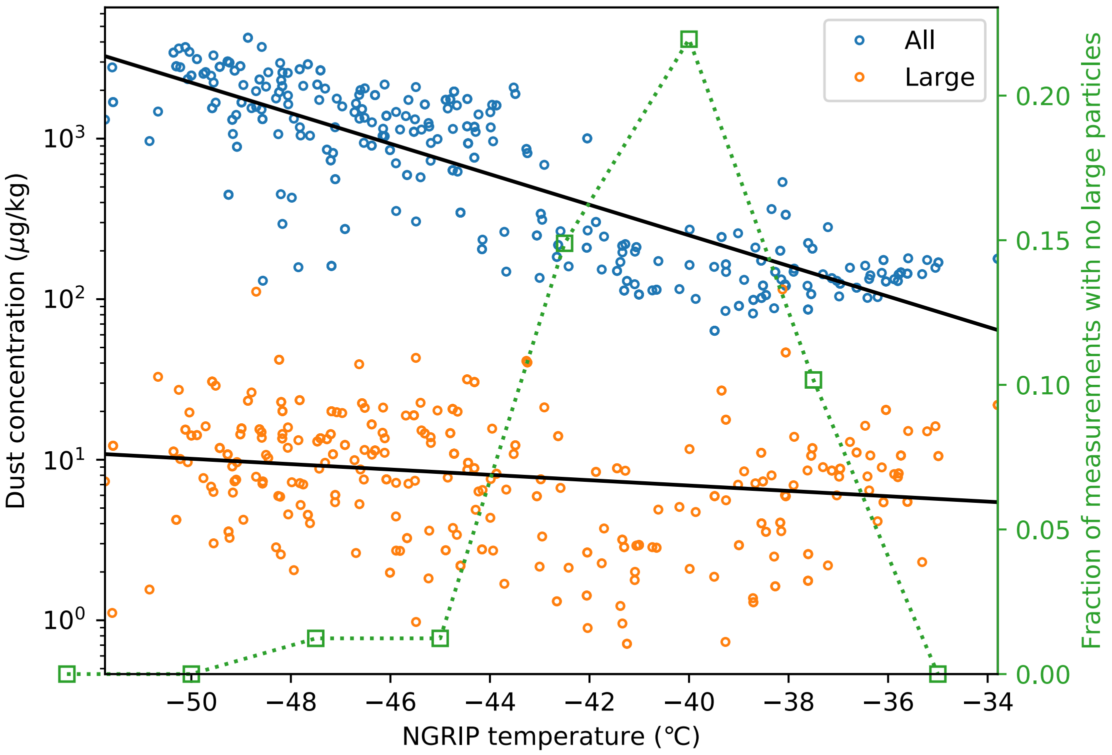

Supplementary Figure 5**: Large glacial particles in the RECAP ice core record.** The orange and blue points are the large particles (>8.13 μm) and full measured size range (1.25-10.5 μm) for the glacial (111.1 - 12.1 ka b2k) as a function of temperature at NGRIP. The black lines are linear fits to the logarithm of the dust concentration. The green line shows the fraction of data points with no large particles in 2.5 °C intervals. The data are downsampled to 200 year intervals.

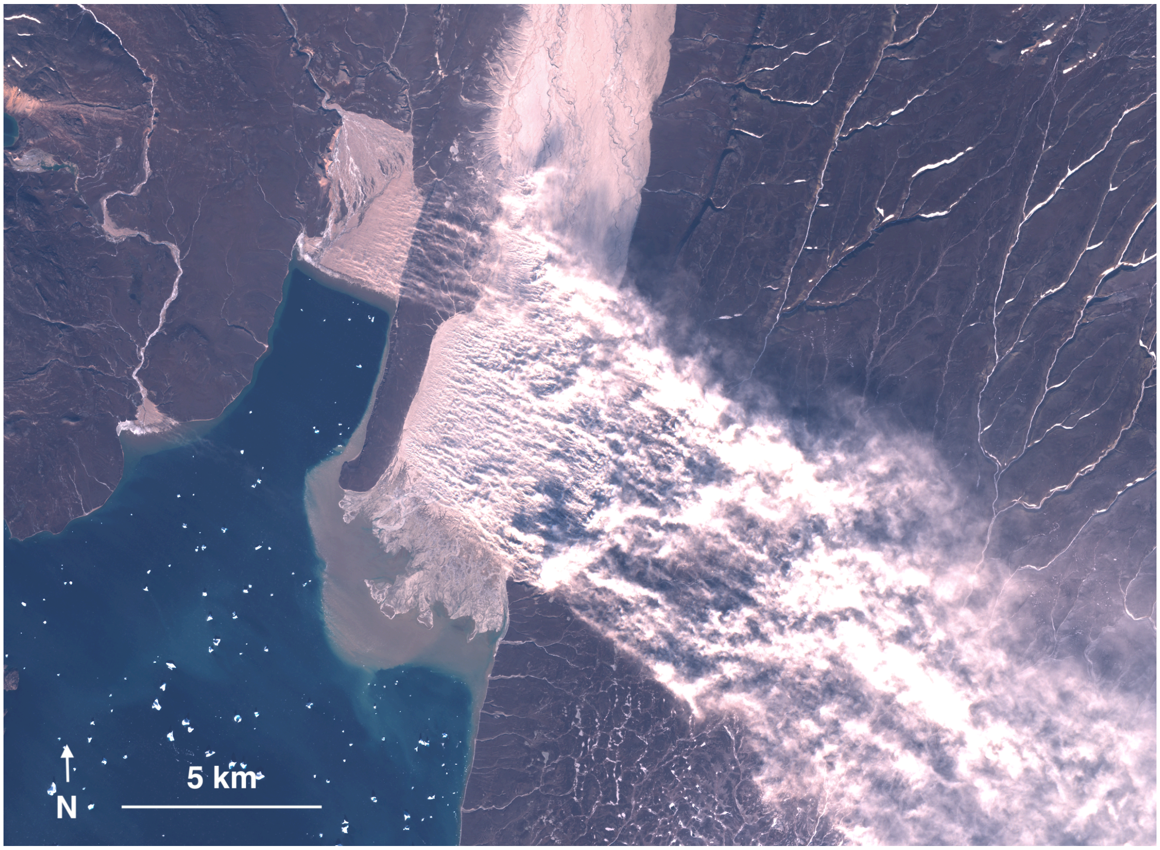

Supplementary Figure 6: **Dust deflation from outwash plains in Schuchert Dal and Gurreholm Dal in Scoresby Sund.** Dust storm captured by the Copernicus Sentinel 2 satellite on September 29 2018. Note the dust is blown to the east, away from Renland ice cap and consistent with down-fjord katabatic winds. Location 71°19'N, 24°33'W. Image credit: Copernicus Sentinel 2 data, processed by ESA, licenced under CC BY-SA 3.0 IGO.

Supplementary Note 1

The RECAP annual-layer counted timescale

The RECAP timescale down to 458.3 m was produced using the StratiCounter software (https://github.com/maiwinstrup/StratiCounter) [2], extended to include multiple chemistry series in parallel with annual signal [3]. StratiCounter was here constrained to fit volcanic marker horizons dated in other Greenland ice cores (DYE-3, GRIP, GRIP and NEEMS1), thereby tying the RECAP timescale to the Greenland Ice Core Chronology 2005 [4]. The upper 10.9 m was manually counted. Below this topmost section, the timescale was derived fully automatically between volcanic markers. The upper 93.5 m of the core has been measured twice by continuous flow analysis, and both measurements have been used with even weight in StratiCounter.

Volcanic match

The synchronization to the GICC05 time scale was carried out below the annual layer counting down to the 8.2 ka cold event by continuing the match of volcanic reference horizons between RECAP and the Central Greenland cores using ECM data. The synchronization procedure followed the methodology used for the Renland core drilled in 1988 outlined in Vinther et al. 2008 [5], i.e. shape-preserving piecewise cubic Hermite interpolation was used between reference horizons.

Synchronization to the Rasmussen et al. 2014 stratigraphy

In Rasmussen et al. 2014 [6] a common stratigraphic framework was developed for the GRIP, GISP2 and NGRIP cores, identifying climatic transitions common to all three cores from the 8.2 ka cold event down through the entire Glacial period. All climatic transitions that were identifiable in the RECAP core were used to create the RECAP time scale for the period from 8.2 ka to 110.6 ka BP. Given the extreme thinning of the RECAP glacial ice, the highly resolved dust measurement was the main parameter used for this synchronization. An example of the impressive resolution provided by the dust as well as the synchronization itself can be seen in Supplementary Figure 1. As there is no systematic flow related thinning of the glacial layers with depth in the RECAP core, the age-depth scale is linearly interpolated between climatic transitions in the glacial period. The Holocene section (8.2 ka to 11.7 ka b2k) is systematically thinning with depth, hence the shape-preserving piecewise cubic Hermite interpolation used in between Holocene volcanic reference horizons was also used here.

Gas Synchronization to NGRIP by CH_4 and $\delta^{18}O_{atm}$ records

The transition from GI-25 to GS-25 dated to 110,640 years b2k in GICC05modelext [6] is the deepest transition clearly identifiable in the RECAP dust record. Hence, below this transition a different dating methodology relying on globally well-mixed atmospheric gasses has been used. Three tie-points constrain the deepest part of the RECAP time scale: (1) the peak in NGRIP CH_4 [7] observed during GI25 (see Supplementary Figure 2), (2) and (3) the sharp change in NGRIP $\delta^{18}O_{atm}$ values [8] observed approximately 120 ka b2k (see Supplementary Figure 2 and Supplementary Figure 3). The RECAP time scale has been linearly interpolated between the three tie points; hereby the difference between

gas ages and ice ages has been ignored for the RECAP gas data. The latter decision is justified by the small delta age during warm climatic periods at Renland. Under present day conditions, the Δage is less than 100 years due to high accumulation rates and melt. The number of $\delta^{18}O_{atm}$ values suitable to serve as tie points is limited in RECAP. They assure that the linear trend in the RECAP $\delta^{18}O_{atm}$ data set exactly matches the linear trend in the NGRIP $\delta^{18}O_{atm}$ data. It should be noted, however, that a number of RECAP $\delta^{18}O_{atm}$ measurements were left out of the RECAP age model as the $\delta^{15}N$ values (measured on the same samples) were too low (less than 0.1) to be consistent with a normal firn column. It is speculated that significant melt during the Eemian warm period took place at Renland causing these low $\delta^{15}N$ values due to melt layers prematurely sealing off the firn column. $\delta^{15}N$ measurements are used to correct for mass dependent fractionation effects (gravitation) in the firn column. Applying the correction to $\delta^{18}O_{atm}$ data with anomalous $\delta^{15}N$ in RECAP leads to inconsistencies. Consequently the values are excluded from further analysis.

It should be stressed that the oldest part of the RECAP time scale is less precisely constrained than the part of the time scale that is based on the dust tie points. This is both due to data gaps caused by small sections of poor core quality (i.e. the gap in the RECAP CH_4 record seen in Supplementary Figure 2) and the large scatter in both RECAP $\delta^{15}N$ and $\delta^{18}O_{atm}$ data. Hence, it cannot be ruled out that the part of the time scale constrained by the RECAP trend in $\delta^{18}O_{atm}$ could contain stratigraphic disturbances both due to excessive melt and possible folds. Despite these uncertainties the RECAP $\delta^{18}O_{atm}$ values do seem to be entirely inconsistent with a younger age for this section of the RECAP core (see Supplementary Figure 3).

Supplementary Note 2

Large glacial particles

During the glacial, the logarithm of the total dust concentration has a -84±2% Pearson correlation with the temperature at NGRIP (Supplementary Figure 5). A linear fit for the logarithm of the dust concentration as a function of NGRIP temperature gives that the dust concentration drops by a factor 28±3 over 15 °C. The large particles have a Pearson correlation by only -25±4% with NGRIP temperature, and drop by a factor 2.2±0.4 over 15 °C . For the fit, only data points with non zero large particles were used. This gives a resolution dependent bias, as lower resolution would have averaged the zeros with the non zero data points, and thereby given a lower value for data points used for the fit. To correct for this bias, the fraction p of zero valued data points for each 2.5 °C interval was calculated (Supplementary Figure 5). All non-zero concentrations in each interval were then multiplied by $(1 - p)$ for the interval. The linear fit was then reapplied to this zero corrected data set, giving a factor 2.6±0.5 per 15 °C instead of 2.2±0.4 for the uncorrected data. The effect of neglecting the zero valued data points is therefore small compared to both the δ^{18}O correlation with large particles and especially the difference between the δ^{18}O correlation with total dust concentration and large particles.

Supplementary Note 3

Possible source areas in the Scoresby Sund region

Strontium and neodymium isotope concentration ratios have been measured in sediment samples collected from the Scoresby Sund region as well as rock powder samples collected by the Danish Geological Survey [9]. Details regarding location and measurement results are provided in Supplementary Table 1.

Supplementary References

1 Kindler, P. *et al.* Temperature reconstruction from 10 to 120 kyr b2k from the NGRIP ice core. *Clim. Past* **10,** 887-902 (2014).
2 Winstrup, M. *et al.* An automated approach for annual layer counting in ice cores. *Clim. Past* **8,** 1881-1895 (2012).
3 Winstrup, M. *A Hidden Markov Model Approach to Infer Timescales for High-Resolution Climate Archives*. (AAAI Press, 2016).
4 Rasmussen, S. O. *et al.* A first chronology for the North Greenland Eemian Ice Drilling (NEEM) ice core. *Clim. Past* **9,** 2713-2730 (2013).
5 Vinther, B. M. *et al.* Synchronizing ice cores from the Renland and Agassiz ice caps to the Greenland Ice Core Chronology,. *J. Geophys. Res.* **113,** D08115, doi:08110.01029/02007JD009143 (2008).
6 Rasmussen, S. O. *et al.* A stratigraphic framework for abrupt climatic changes during the Last Glacial period based on three synchronized Greenland ice-core records: refining and extending the INTIMATE event stratigraphy. *Quat. Sci. Rev.* **106,** 14-28 (2014).
7 Baumgartner, M. *et al.* NGRIP CH_4 concentration from 120 to 10 kyr before present and its relation to a $\delta^{15}N$ temperature reconstruction from the same ice core. *Clim. Past* **10,** 903-920 (2014).
8 Capron, E. *et al.* Synchronising EDML and NorthGRIP ice cores using $\delta^{18}O$ of atmospheric oxygen ($\delta^{18}O_{atm}$) and CH_4 measurements over MIS5 (80-123 kyr). *Quat. Sci. Rev.* **29,** 222-234 (2010).
9 Rex, D. C. & Gledhill, A. R. Isotopic studies in the East Greenland Caledonides (72°-74°N) - Precambrian and Caledonian ages. *Rapport Grønlands Geologiske Undersøgelse* **104,** 47-72 (1981).
